# Vanadium Modulates Proteolytic Activities and MMP-14-Like Levels during *Paracentrotus lividus* Embryogenesis

**DOI:** 10.3390/ijms232214238

**Published:** 2022-11-17

**Authors:** Roberto Chiarelli, Chiara Martino, Rosaria Scudiero, Fabiana Geraci

**Affiliations:** 1 Department of Biological, Chemical and Pharmaceutical Sciences and Technologies (STEBICEF), University of Palermo, Viale delle Scienze Building 16, 90128 Palermo, Italy; 2 Department of Biology, University Federico II, 80126 Napoli, Italy

**Keywords:** vanadium, sea urchin embryos, morphology, morphometry, gelatinases, metalloproteinases, MMP-14

## Abstract

The increasing industrial use of vanadium (V), as well as its recent medical use in various pathologies has intensified its environmental release, making it an emerging pollutant. The sea urchin embryo has long been used to study the effects induced by metals, including V. In this study we used an integrated approach that correlates the biological effects on embryo development with proteolytic activities of gelatinases that could better reflect any metal-induced imbalances. V-exposure caused morphological/morphometric aberrations, mainly concerning the correct distribution of embryonic cells, the development of the skeleton, and the embryo volume. Moreover, V induced a concentration change in all the gelatinases expressed during embryo development and a reduction in their total proteolytic activity. The presence of three MMP-like gelatinases (MMP-2, -9, and -14) was also demonstrated and their levels depended on V-concentration. In particular, the MMP-14-like protein modified its expression level during embryo development in a time- and dose-dependent manner. This enzyme also showed a specific localization on filopodia, suggesting that primary mesenchyme cells (PMCs) could be responsible for its synthesis. In conclusion, these results indicate that an integrated study among morphology/morphometry, proteolytic activity, and MMP-14 expression constitutes an important response profile to V-action.

## 1. Introduction

Vanadium (V) is a trace element with a wide diffusion among living organisms and in different environments, as it is present in the soil, air, and water [1,2,3]. It represents an emerging environmental pollutant as it is an industrial waste in metallurgical preparations and the combustion of crude or residual oil and coal [1]. In recent years, V-complexes have been considered as a possible new class of metallodrugs [4,5,6], potentially increasing V environmental waste. The release of medicinal products for human use into the aquatic environment is nowadays a serious problem and can be fatal for the organisms that live there [7].

Therefore, the current central problem is, on one hand, to control V-release into the environment and, on the other hand, considering the possible therapeutic applications for its compounds, to refer to environmental bioindicators that can act as pollution sentinels. Nevertheless, the study of cell signaling pathways related to V-compounds has scarcely been reported [8]. This information could be highly critical to identify novel targets that could play a key role in the therapeutic activity of these compounds. In parallel, it could be important for selecting molecular markers in bioindicator organisms. It is now well known that the toxicity and bioavailability properties of an element are highly dependent upon its chemical form. Therefore, to completely understand the biochemical impact of elements on living organisms, it is essential to consider metal speciation. It has been reported that the chemical nature of the tested metal compounds could change depending on cell culture conditions [9].

Here, we have performed research on V-activity over cell response in an embryonic model system, which has already been standardized for studies on the effects triggered by different metals, including V [3,10,11,12,13,14,15,16,17,18,19,20,21,22,23].

Sea urchin embryos (*Paracentrotus lividus*) represent one of the few well-established marine biological systems that have been applied in several studies because of their sensitivity to many emerging contaminants [24,25,26]. The sea urchin embryo also exemplifies a model for studying molecular mechanisms involved in human diseases, as well as for testing bioactive compounds [27]. The potential of its use as a model for disease research relies on the fact that general cellular pathways are common among many organisms. The complete sequencing of its genome has also revealed that sea urchins are more closely related to humans than other invertebrates [28,29]. Sea urchin embryos offer the advantage of having no excretion mechanisms, thus accumulating the tested substances [13].

Previous studies conducted on sea urchin embryos reported that V induced developmental delays and anomalies, especially in the skeleton. Biomineralization appeared to be inhibited as the amount of calcium (Ca) present in V-treated embryos was drastically reduced [23]. V induced a stress response mediated by heat shock proteins (i.e., HSP60 and HSP70), and by massive autophagic processes [3]. During embryogenesis, V bioaccumulated exponentially and modulated the activation of the ERK pathway. Finally, in the late stages of development (i.e., early and advanced pluteus), it caused a cell-selective apoptosis [23]. Regarding the activation of proteolytic activities, it was observed that V at different cytotoxic/environmental concentrations triggered the modulation of nine proteases at the pluteus stage (36 h of treatment) [22].

Proteases in sea urchin play a key role during embryogenesis, a process that requires the formation of new structures and drastic changes in the organization of cells, tissues, and organs. The expression of these enzymes is finely regulated, and their action must be carefully managed in space and time in such a way as to allow the complete development of the dynamic structure of the embryo. These enzymatic activities play an important role in tissue remodeling, cell migration, differentiation, and morphogenesis [22,30].

The aim of this work was to evaluate the potential relationship between the morphological/morphometric effects induced by V and the modulation of gelatinase activity during embryonic development.

These proteolytic activities include enzymes whose function depends on metal ions used as cofactors. Some of these enzymes are metalloproteases (MMPs), a family of zinc (Zn)- and Ca-dependent proteinases, that are regulated by V in different organisms [31,32,33,34].

MMPs are necessary for multiple and diverse physiological processes, such as reproduction, morphogenesis, embryonic development, bone remodeling, angiogenesis, and tissue repair, but they can also contribute to tissue destruction during cancer development and spreading, in arthritis/osteoarthritis and in fibrotic diseases [35].

Among members of the MMP family, MMP-14 belongs to a subgroup that presents a transmembrane domain, with a cytosolic tail and the catalytic site exposed in the extracellular space [36]. Previous studies have highlighted its pleiotropic functions, especially in the regulation of developmental events requiring extracellular matrix remodeling, such as cell polarization and migration, during embryogenesis [37,38,39,40,41]. MMP-14 involvement in cell migration is confirmed by its localization on lamellipodia, filopodia, and invadopodia of migrating cells [42,43,44,45]. The important role of this enzyme during embryo development is demonstrated in deficient mice. Its absence results in early postnatal death and displays severe defects in skeletal, muscle, and lung development [36].

However, despite that MMP-14 plays critical roles in development, little is known about its expression pattern in invertebrate embryos and its modulation due to V-exposure. For this reason, the variable expression of an MMP-14-like protein has been studied both during physiological sea urchin development and following V-treatment.

In conclusion, our work suggests that the integrated study of morphology/morphometry, with the levels of proteolytic activities and the expression of an MMP-14-like protein, constitutes an important response profile to the action of V in this embryonic experimental system.

## 2. Results

### 2.1. V Induces Morphological and Morphometric Effects during Sea Urchin Embryogenesis

**Figure 1:**
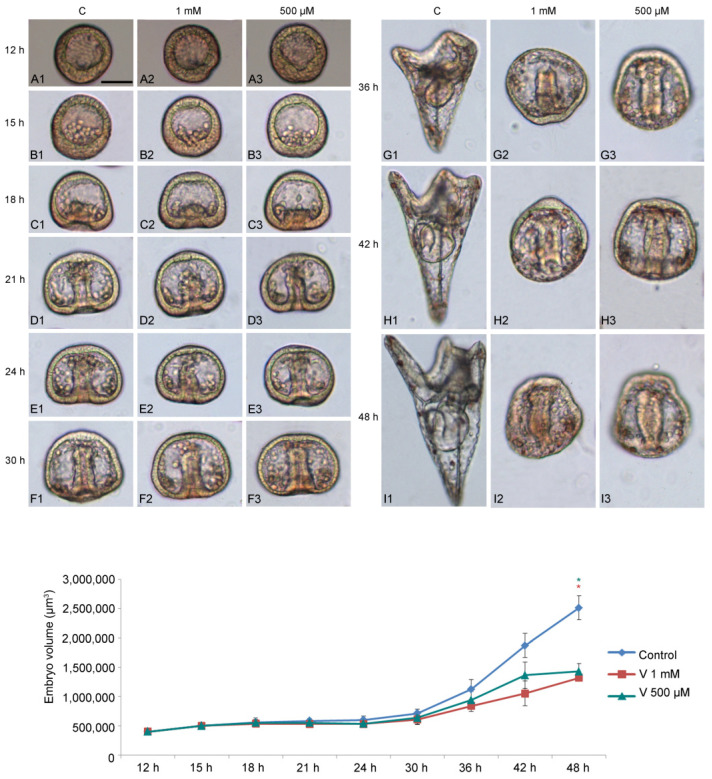
Morphological and morphometric effects induced by V during embryonic development. Images representative of embryos from 12 h to 48 h of development/treatment. (**A1**,**B1**,**C1**,**D1**,**E1**,**F1**,**G1**,**H1**,**I1**) Control embryos; (**A2**,**B2**,**C2**,**D2**,**E2**,**F2**,**G2**,**H2**,**I2**) 1 mM V-treated embryos; (**A3**,**B3**,**C3**,**D3**,**E3**,**F3**,**G3**,**H3**,**I3**) 500 µM V-treated embryos. Control and V-treated embryos were observed at different development/treatment intervals: (**A1**–**A3**) 12 h; (**B1**–**B3**) 15 h; (**C1**–**C3**) 18 h; (**D1**–**D3**) 21 h; (**E1**–**E3**) 24 h; (**F1**–**F3**) 30 h; (**G1**–**G3**) 36 h; (**H1**–**H3**) 42 h; and (**I1**–**I3**) 48 h. Scale bar = 45 µm. Images are representative of three independent experiments. The line graph shows data related to the volumetric analysis. Experiments were performed in triplicate and data are expressed as means ± standard deviation (*n* = 3 ± SD). Statistical analysis was performed using *t*-test with * *p* ≤ 0.05.

To study how V affects embryo development, we used two different metal concentrations (1 mM and 500 µM) and examined their effects during sea urchin embryogenesis, starting from 12 h to 48 h of development (Figure 1A–I). We focused our attention on the following developmental stages: HB (hatching blastula, 12 h); MB (mesenchyme blastula, 15 h); EG (early gastrula, 18 h); IG (intermediate gastrula, 21 h); AG (advanced gastrula, 24 h); Pr (prism, 30 h); EPl (early pluteus, 36 h); and APl (advanced pluteus, 42–48 h).

V-exposure was well tolerated in the earliest stages of development (HB, MB, and EG) (Figure 1(A1–C3)), with a continuous exposure until later stages, initially caused a delay in development and subsequently induced embryo anomalies, represented by morphological and volumetric alterations.

Compared with controls, embryos treated with 1 mM showed a significant developmental delay, starting from 21 h of treatment (Figure 1(D1,D2)). As the development progressed (from 30 h to 36 h), when the control embryos reached the Pr or EPl stage (Figure 1(F1–G1)), V-treated embryos showed morphological anomalies, essentially related to the arrangement of the primary mesenchyme cells (PMCs) (Figure 1(F2,F3,G2,G3)).

From this stage onwards (42–48 h), V-exposed embryos showed a total deficiency of the skeleton, indicative of an absence of biomineralization that, in sea urchin embryos, corresponds to the deposition of calcium and magnesium ions, a scarce elongation of the archenteron, and an abnormal embryo symmetry (Figure 1(H1–I3)).

During development, control embryos showed a five times increase in their volume compared with the initial stages (Figure 1 line graph). This quantitative parameter is an indicator of correct development, as a volumetric increase occurs as a result of the expansion of the blastocoel cavity, the elongation of the archenteron, and the formation of the spicules. Although V-treated embryos developed the blastocoelic cavity and elongated the archenteron, both were poorly expanded. The main reason that led to the failure to increase the volume observed from 30 h of development was the total absence of the skeleton. In this condition, no volumetric increase sustained by the growth of the spicules was observed.

As shown in Figure 1 line graph, embryos exposed to both V-concentrations exhibited less growth (i.e., 2–3 times) than in the initial stages of development.

Quantitative analysis related to morphological observations, reporting delays and anomalies for each developmental stage, is described in Table 1.

As expected from our previous works, V had a significant effect on the progression through developmental stages in *P. lividus* embryos (F26, 54 = 377.33, *p* < 0.0001), as well as its interaction with time (ANOVA time × V: F26, 54 = 460.71, *p* < 0.0001). Pair-wise tests (Tukey HSD) revealed significant differences between the 27 different treatments in the percentage of abnormal embryos with respect to controls of the same stage from 21 h, while no differences were present at earlier stages (Table 1).

It was possible to observe that, when control embryos were at HB and MB stage (12 h and 15 h respectively), treated embryos did not show any morphological change, indicating that, up to these stages, they were able to tolerate both V concentrations. After 18 h of treatment (i.e., EG stage), a not significant percentage of developmental delays (3% MB) began to appear in 1 mM V-exposed embryos. Moving forward with development (21 h, IG stage), embryos exposed to both V-concentrations showed significant growth delays (98% for 1 mM and 50% for 500 µM V-exposed embryos). Despite these delays, development never stopped, and embryos survived without evident morphological anomalies up to 30 h of V-treatment. Conversely, at 36 h (EPl stage), V-treated embryos displayed not only developmental delays, having an AG or Pr-like phenotype, but also morphological anomalies with the complete abolition or drastic reduction in the biomineralization process, showing no skeletal formation, the abnormal migration of embryonic cells, a reduction in embryo growth and in archenteron length, and the lack of embryo symmetry. In particular, at 36 h 1 mM, exposed embryos were 100% abnormal AG, while those exposed to V 500 µM were 98% abnormal AG and only 2% were normal Pr. This trend of aberrations was also observed at 42 h and 48 h of exposure. In all cases, these embryos showed serious morphological anomalies, especially regarding skeletogenesis.

### 2.2. V and Ca Accumulation Correlates with Treatment Doses

**Figure 2:**
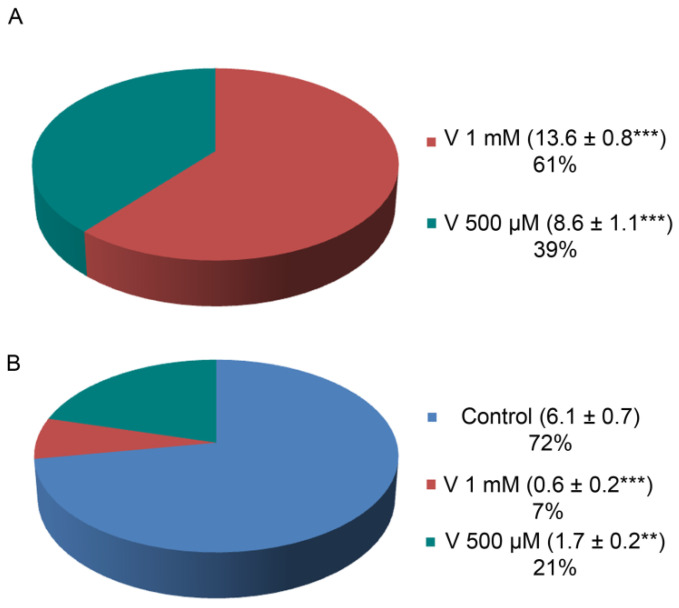
Amount of V and Ca incorporated in embryos after 48 h of development/treatment. Embryos were cultured under normal growth conditions and with V 1 mM or 500 µM. (**A**) V and (**B**) Ca content. Values reported in the pie charts show the ionic concentrations detected for V (µg/g weight embryos) and Ca (mg/g weight embryos). Experiments were performed in triplicate and data are expressed as means ± standard deviation (*n* = 3 ± SD). Statistical analysis was performed using *t*-test with ** *p* ≤ 0.01; *** *p* ≤ 0.0005.

It has already been reported that V-accumulation in sea urchin embryos caused Ca depletion, ERK modulation, and the activation of a cell-selective apoptosis [23]. Here, in order to detect a possible correlation between the levels of both ions and the presence of proteolytic activities, we have first detected the internal ion concentration in embryos at the final development/treatment stage (48 h). This determination allows to consider only the quantity actually incorporated by embryos, excluding the ions lost during development because of cell-selective apoptosis. V and Ca content were detected by inductively coupled plasma mass spectrometry (ICP-MS). Embryos absorbed a different amount of V depending on the used dose (Figure 2A). Specifically, embryos exposed to 1 mM V accumulated a quantity of this ion equal to 1.6 times compared with embryos exposed to 500 µM V. At the same time, Ca accumulation was reduced in V-exposed embryos compared with controls, and the reduction was markedly higher (about one-third) in embryos treated with 1 mM V than in those exposed to 500 µM V (Figure 2B).

### 2.3. Total Proteolytic Activity Depends on Treatment Time and V-Concentration

As previously described by Chiarelli et al., V can influence sea urchin embryos’ development promoting, at the pluteus stage, a variation in metal-related proteolytic activities [22]. These proteases are active throughout the entire embryonic development, and their activity is essential for correct growth [46,47,48]. For this reason, we tested the proteolytic activity in lysates of control and V-treated embryos obtained from blastula up to pluteus.

Using gelatin substrate zymography, a dynamic picture of cleavage was identified. Initially, we focused our attention on total proteolytic activity (from 12 h to 48 h of development/treatment). Later, for the same intervals, the single enzymatic activities (309, 255, 177, 79, 59, 34, 30, 25, and 22 kDa respectively, as already described by Chiarelli et al.), were analysed [22].

**Figure 3:**
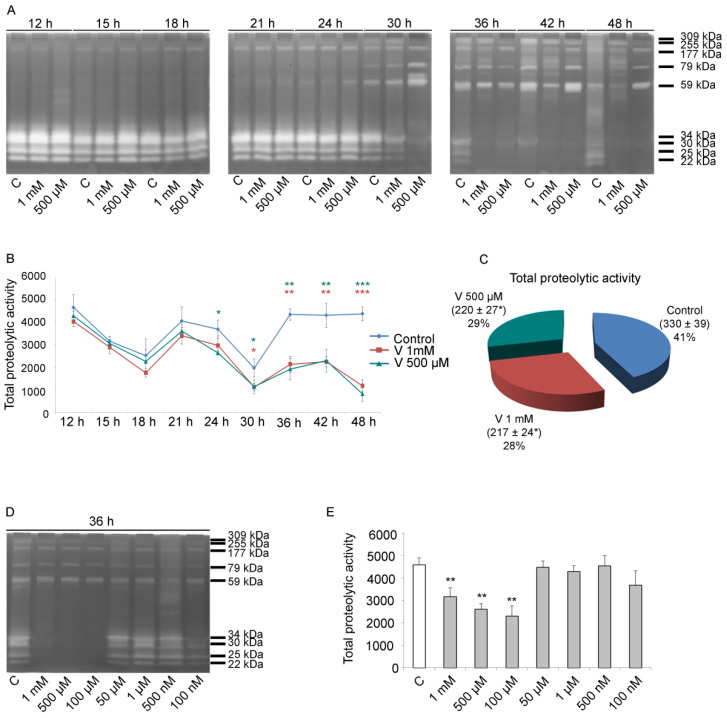
Metal-related enzymatic activities analysed by gelatin substrate gel zymography. (**A**) Zymograms showing bands in lysates of control and V-treated (1 mM, 500 µM) embryos from 12 h to 48 h of development/treatment. (**B**) Line graph reporting the modulation over time of the total proteolytic activity for each development stage and V-concentration. (**C**) Pie chart showing the distribution of the total proteolytic activity for the entire time of development/treatments. (**D**) Zymogram showing gelatinase bands in lysates of control and V-treated (1 mM; 500 µM; 100 µM; 50 µM; 1 µM; 500 nM; 100 nM) embryos at 36 h of development. (**E**) Histogram showing the total proteolytic activity of control and V-treated (1 mM; 500 µM; 100 µM; 50 µM; 1 µM; 500 nM; 100 nM) embryos at 36 h of development. Experiments were performed in triplicate and data are expressed as means ± standard deviation (*n* = 3 ± SD). The band intensity of panels A and D was measured by Quantity One software. Statistical analysis was performed using *t*-test with * *p* ≤ 0.05; ** *p* ≤ 0.01; *** *p* ≤ 0.0005.

In general, the early embryonic stages were characterized by the activity of the low molecular weight proteolytic enzymes (from 34 kDa to 22 kDa), whereas the late stages showed elevated levels of the high molecular weight proteases (from 309 kDa to 59 kDa) (Figure 3A).

Quantitative analysis of the total proteolytic activity (both low and high molecular weight proteases) indicated that, from 12 h to 30 h, there was a reduction in the levels of these enzymes compared with controls, in embryos exposed to both V concentrations. However, although proteolytic activity was reduced, the trend (increase/decrease during development) was comparable to that of the controls, i.e., a reduction from 12 h to 18 h of development/treatment; an increase at 21 h; and then, again, a reduction at 24 h and 30 h. Otherwise, in the period ranging from 30 h to 48 h of treatment, a drastic and significant reduction in total proteolytic activity was observed in V-exposed embryos. In fact, in this specific time interval, the total proteolytic activity was halved (Figure 3B). Altogether, for the entire development/treatment period, the total proteolytic activity was reduced compared with controls by 35% in embryos exposed to 1 mM V and by 33% in those exposed to 500 µM V (Figure 3C).

As a dose–response effect was observed, to highlight the modulation of the response, experiments increasing the V-concentration spectrum were carried out. In addition to the former V-doses (i.e., 1 mM and 500 µM), we tested further lower doses, ranging from 100 µM to 100 nM, and the effects at 36 h of treatment were examined. The obtained data showed a dose-dependent effect. Embryos modulated their response in terms of total proteolytic activity in the 1 mM–100 µM concentration range, while they were able to well tolerate the lowest V-doses, ranging from 50 µM to 100 nM (Figure 3D,E).

Our results showed that V induced a dose-/time-dependent modulation in the global gelatinase activity.

### 2.4. Single Proteolytic Activity Varies Depending on V-Exposure

**Figure 4:**
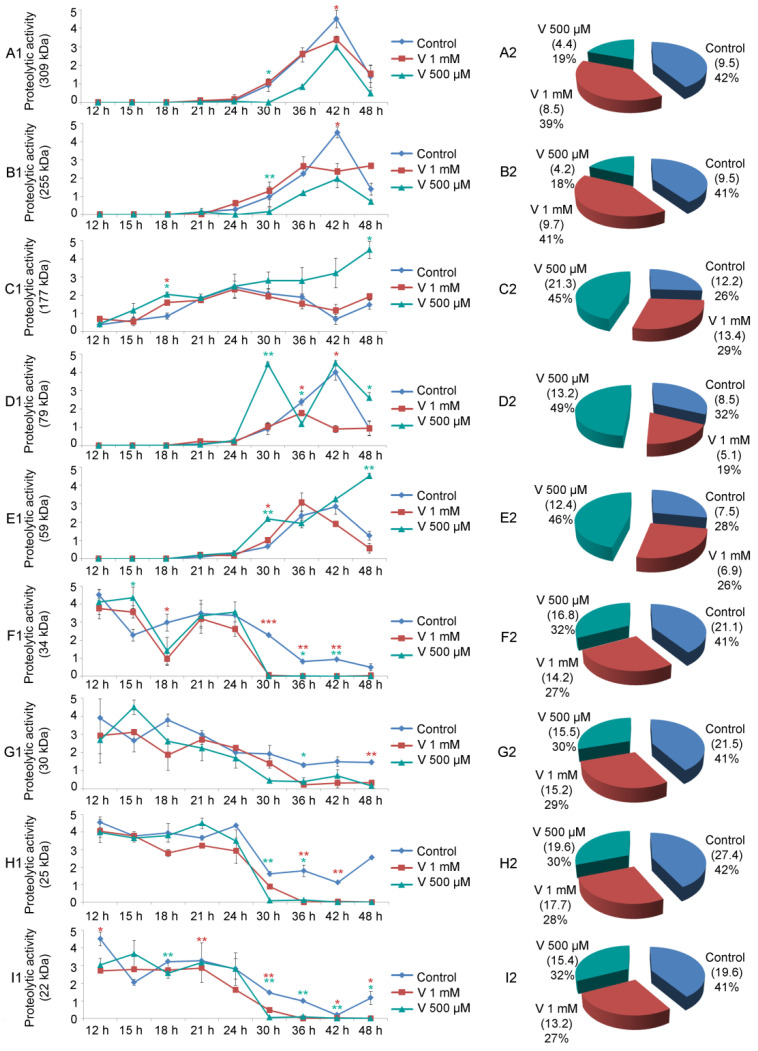
Single proteolytic activities. Line graph reporting the modulation over time, from 12 h to 48 h of each proteolytic activity, in controls and V-treated (1 mM, 500 µM) embryos. According to their molecular weight, the identified proteases are as follows: (**A1**,**A2**) 309 kDa; (**B1**,**B2**) 255 kDa; (**C1**,**C2**) 177 kDa; (**D1**,**D2**) 79 kDa; (**E1**,**E2**) 59 kDa; (**F1**,**F2**) 34 kDa; (**G1**,**G2**) 30 kDa; (**H1**,**H2**) 25 kDa; and (**I1**,**I2**) 22 kDa. The pie charts display the value of each gelatinase activity related to the whole development/treatment time. Experiments were performed in triplicate and graph data are expressed as means ± standard deviation (*n* = 3 ± SD). Statistical analysis was performed using *t*-test with * *p* ≤ 0.05; ** *p* ≤ 0.01; *** *p* ≤ 0.0005.

As already demonstrated by Chiarelli et al., zymography assays confirmed the presence of nine distinct proteolytic activities in sea urchin embryos [22]. Here, we have also showed that these proteases appeared to be differently modulated both during development and by the two different V-treatments (Figure 4A–I).

The 309 kDa and 255 kDa proteases started to show their physiological activity at AG stage (24 h of development), then there was an increase in their levels until the APl stage (42 h of development), followed by a reduction at 48 h of development. For V-treated embryos, although the trend reflected that of the control, the global levels of these two proteases were lower. Specifically, embryo exposure to 500 µM V caused a greater reduction in protease activity than the higher concentration (Figure 4(A2,B2)).

A different behaviour was observed for the 177 kDa protease. It began to be noticeable already during early stages of development (i.e., MB stage, 15 h). Its levels underwent small fluctuations during physiological development and in embryos exposed to 1 mM V. On the other hand, in embryos exposed to 500 µM V, an increased level of this proteolytic activity started from 24 h of treatment, reaching a maximum at 48 h (Figure 4(C1)). Globally, the proteolytic activity of the 177 kDa gelatinase, throughout the development/treatment period, was lower in the control embryos than in both V-treated ones, where it increased (Figure 4(C2)).

The 79 kDa protease showed a peculiar trend. Its activity started in control and V-treated embryos around 24–30 h, when control embryos were between the AG and Pr stage. In control embryos, the levels of this proteolytic activity continued to increase up to the advanced stage of pluteus (42 h). Its levels subsequently decreased at 48 h of physiological development. Embryos exposed to 500 µM V, after a peak at 30 h of treatment, decreased its level at 36 h. It started to rise again until 42 h and remained high up to 48 h of treatment. On the contrary, embryos exposed to 1 mM V displayed a weak peak at 36 h, then its level remained low (Figure 4(D1)). The total level of this gelatinase, for the entire period of development/treatment, was very high in embryos exposed to 500 µM V, whereas it was comparable in control and 1 mM V-exposed embryos (Figure 4(D2)).

The 59 kDa gelatinase started its activity after 24 h of development/treatment (when control embryos reached the AG stage). Its level increased up to 36–42 h. Subsequently these levels decreased in controls and in 1 mM V-treated embryos. The levels of this enzymatic activity, on the other hand, increased up to 48 h of treatment in embryos exposed to 500 µM V (Figure 4(E1)). The activity of this gelatinase throughout the whole development/treatment period, similarly to what was observed for the 177 kDa and 79 kDa, remained higher in embryos exposed to the lowest V-concentration (500 µM) (Figure 4(E2)).

The group of low molecular weight proteolytic activities (34, 30, 25, and 22 kDa) behaved in the same way. Specifically, the trend of these enzymatic activities followed the path observed during physiological development, but their activity levels changed. In general, they were mainly active during the first phases of the embryonic development/treatment (from 12 h to 30 h). In the second part of embryonic development/treatment (from 36 h to 48 h), their levels remained low (Figure 4(F1,G1,H1,I1)). Overall, for this group of proteases, the proteolytic activity levels were high in control embryos and reduced in embryos exposed to both V-concentrations (Figure 4(F2,G2,H2,I2)).

### 2.5. Metalloproteinases-2, -9, and -14-Like Proteins Are Present in Sea Urchin Embryos

The above reported data related to gelatinases have shown how the activity of these enzymes was modulated during the physiological development and in V-exposed embryos. However, zymography assays did not allow us to determine whether there were any similarities with human MMPs.

A previous biochemical characterization showed that the 79 kDa and 59 kDa gelatinases requires Zn^2+^ and Ca^2+^ to maintain their catalytic activity, and their cleavage activity was limited to denatured collagen (gelatin). These data demonstrated that these enzymes are members of the MMP family [22]. Because of this similarity with MMPs, we performed immunoblot analysis using antibodies against mammalian MMP-2, MMP-9, and MMP-14 to determine whether there were similar proteins in sea urchin embryos and whether there was a modulation of these specific metalloproteases in protein levels during development/treatment.

**Figure 5:**
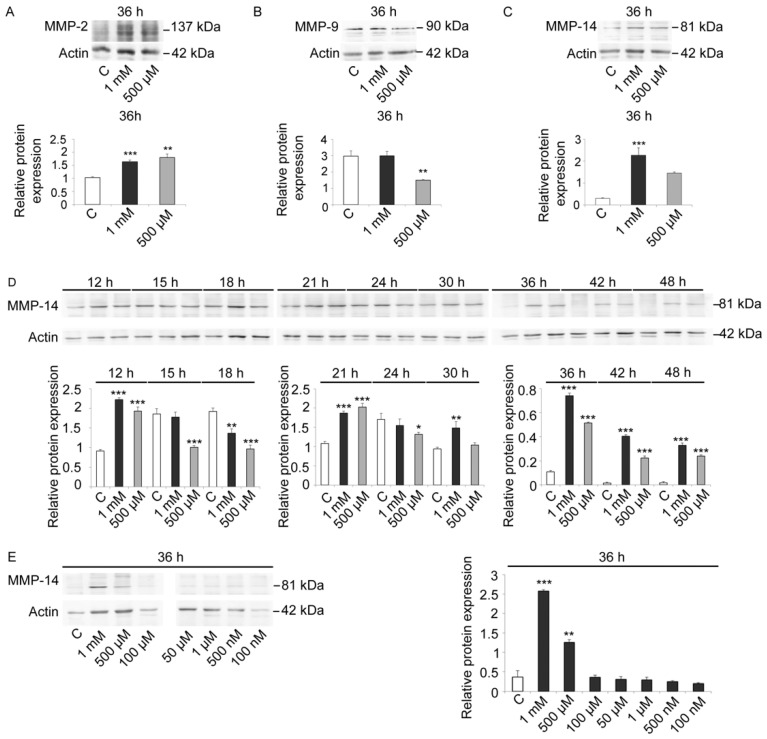
Immunoblotting detection and quantitative analysis for MMPs. (**A**–**C**) Total lysates of control and V-treated embryos (1 mM, 500 μM) after 36 h of development/treatment, immunoreacted with anti- (**A**) MMP-2, (**B**) MMP-9, and (**C**) MMP-14 antibodies. Histograms showed the densitometric analysis of the obtained bands. (**D**) Total lysates of control and V-treated embryos (1 mM, 500 μM) after 12, 15, 18, 21, 24, 30, 36, 42, and 48 h of development/treatment immunoreacted with anti-MMP-14 antibodies. Histograms showed the densitometric analysis of the bands. (**E**) Total lysates of control and V-treated (1 mM; 500 µM; 100 µM; 50 µM; 1 µM; 500 nM; 100 nM) embryos after 36 h of development/treatment immunoreacted with anti-MMP-14 antibodies. Histograms showed the densitometric analysis of the bands. Actin was used as a loading control. Relative protein expression, reported as arbitrary units, was calculated as the band density ratio to that of actin. Experiments were performed in triplicate and data are expressed as means ± standard deviation (*n* = 3 ± SD). Statistical analysis was performed using *t*-test with * *p* ≤ 0.05; ** *p* ≤ 0.01; *** *p* ≤ 0.0005.

As these antibodies were constructed for mammalian enzyme isoforms, we first evaluated the signal specificity and the variations in control and V-treated embryos at 36 h, when control embryos were at EPl stage (Figure 5A–C). All three antibodies reacted with sea urchin embryo lysates, suggesting the presence of MMP-2-, MMP-9-, and MMP-14-like proteins. Based on this analysis, we also observed that MMP-14 showed the greatest modulation in response to V-treatment (Figure 5C). Moreover, as a well-documented role in embryogenesis and skeletogenesis was reported for this MMP, we focused our attention on it [37,49,50,51,52].

Immunoblot analysis with anti-MMP-14 antibodies carried out on lysates of control and V-treated embryos (1 mM and 500 µM) at different time points showed that an MMP-14-like protein was already physiologically expressed at HB stage (Figure 5D). An increase in its levels was observed at MB and EG stages. Later on, protein levels remained generally constant under physiological growth conditions. Only a weak increase was observed at AG stage, whereas a drastic reduction was observed at 30 h and 36 h of development (Pr and EPl stages, respectively). A faint signal was detected in control embryos at 42 h and 48 h (APl stages).

In embryos treated with both V-concentrations (1 mM or 500 µM), a high level of MMP-14 was observed, with a dose-dependent response (Figure 5D).

After 12 h of treatment, embryos showed an increased MMP-14-like level over control equal to 2.4 and 2.1-fold, respectively. On the other hand, after 15 h of treatment, V-treated embryos showed a gradual reduction in MMP-14 amount. This reduction markedly occurred after 18 h of treatment and was about 30% for embryos exposed to 1 mM V and about 50% for embryos exposed to 500 µM V. At the initial gastrulation phase (21 h of treatment), there was a very significant increase in MMP-14-like levels (about 1.8-fold for both V-treatments). On the contrary, at 24 h of treatment, the level of MMP-14-like protein decreased slightly for both V-treated embryos. At 30 h of treatment, the levels increased again, and, at 36 h, they continued to increase for both concentrations (5.8- and 4.7-fold). This increase persisted in a dose-dependent manner at 42 h and 48 h of V induction (histograms in Figure 5D).

To better investigate the link between the dose-dependent V-effects and the expression levels of the MMP-14-like protease, the V-concentration range has been expanded. In addition to the two already used V-doses (i.e., 1 mM and 500 µM), we tested lower V doses, from 100 µM to 100 nM, and we observed the effects after 36 h of treatment. The obtained data showed the presence of a dose-dependent effect and a certain tolerance level. Embryos were able to tolerate the lowest doses of V (100 µM–100 nM), as the observed MMP-14-like levels were comparable to control. Similarly to what observed for the total proteolytic activities, embryos were able to modulate the response in terms of MMP-14-like levels only for the high V-concentrations (1 mM and 500 µM) (Figure 5E).

### 2.6. Localization of MMP-14 in Sea Urchin Embryos Showed Peculiar Expression Districts

Further analysis carried out by immunofluorescence assays with anti-MMP-14 antibodies showed a specific localization of this enzyme.

We focused our attention on 36 h of development/treatment, when control embryos were at EPl stage. This choice was because of the greatest variation in MMP-14-like expression level among control and V-treated embryos (Figure 5D). At this stage, we also found the most considerable variations induced by the metal at the morphological/morphometric level. Indeed, V-exposed embryos showed an arrest in biomineralization and a delayed phenotype (Figure 1(G1–G3)).

**Figure 6:**
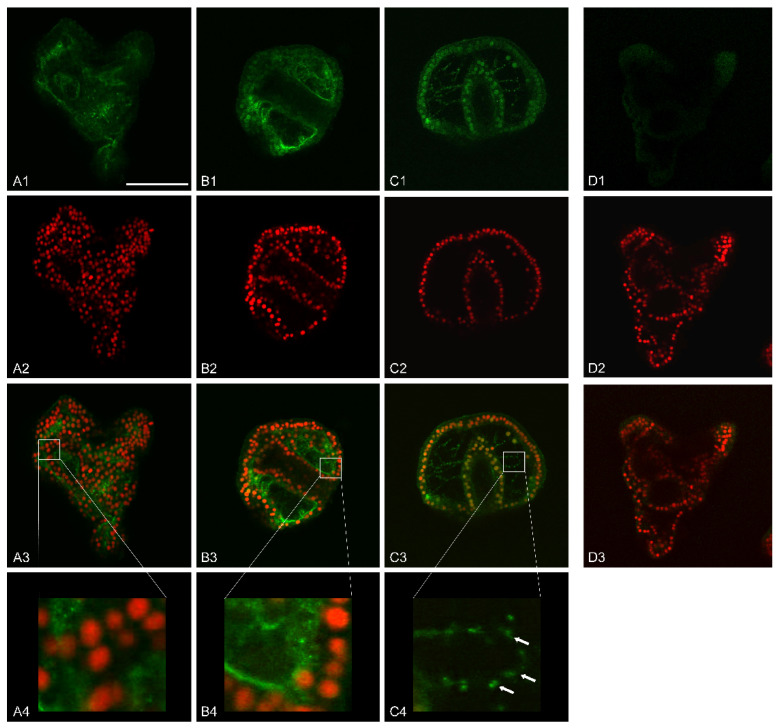
Immunolocalization analysis of MMP-14-like protease in whole mount embryos. Shown are representative confocal microscopy images of equatorial optical sections of embryos at 36 h of development/treatment. (**A1**,**B1**,**C1**) MMP-14-like protein detection; (**A2,B2,C2,D2**) nuclei counterstaining with propidium iodide (PI); (**A3**,**B3**,**C3**,**D3**) merge of green (MMP-14) and red (PI) signals. (**A1**–**A3**) Control embryo; (**D1**) negative control with only the secondary antibody; (**B1**–**B3**) 1 mM V-treated embryo; (**C1**–**C3**) 500 µM V-treated embryo. (**A4**,**B4**,**C4**) Higher magnification of a section of (**A3**) control, (**B3**) 1 mM V-treated, and (**C3**) 500 µM V-treated embryo, respectively. White arrows indicate the localization of MMP-14-like in filopodia. Scale bar = 45 µm.

Immunofluorescence analysis by confocal laser scanning microscopy (CLSM) highlighted that control embryos had a basal MMP-14-like signal, with a cytoplasmic localization. The most affected embryonic areas were represented by the wall of both the mouth and stomach, the apical end, the pre-oral arms, and part of the ectoderm (Figure 6(A1–A4)).

Embryos exposed to 1 mM V showed a predominantly perinuclear signal in the outer portion of the archenteron and in the inner portion of the blastocoel. In this area, the signal was present partly on thin protrusions, filopodia-like, adhering to the blastocoelic wall and, partly, in thin filaments, probably cytoplasmic cords, that connected the outer part of the archenteron to the inner wall of the blastocoel cavity (Figure 6(B1–B3)).

On the other hand, embryos treated with V 500 µM had a more defined MMP-14 localization. The signal was predominantly perinuclear and affected all three embryonic leaflets, ectoderm, mesoderm, and endoderm. Moreover, in this case, filopodia-like processes connecting the outer wall of the archenteron to the inner wall of the blastocoel cavity were observed. These filamentous protrusions also showed a left/right symmetry in the embryos. The MMP-14-like signal in these filamentous structures appeared in the form of granular clusters arranged in a row, one behind the other. Furthermore, CLSM analysis showed that some of these filamentous projections branched off from the PMCs and, globally, formed a scaffold within the blastocoel (Figure 6(C1–C4)).

## 3. Discussion

The sea urchin embryo represents an interesting and standardized model system for studies aiming to identify the biological effects induced by metals, of both environmental and industrial/pharmaceutical interest. This organism is not subject to the animal welfare legislation, allowing a decrease in the use of classical laboratory animals. Considered the most primitive deuterostome, sea urchin is phylogenetically correlated to both protochordates and vertebrates [53,54]. This bioindicator model offers the advantage to investigate the metal-related response in the whole organism, in which cells interact in their physiological position [3].

V is becoming an emerging environmental pollutant because of its growing interest in the industrial and biomedical field, responsible for its increasing environmental release, making studies about its effects on marine life essential. In this paper, we investigated the V response in sea urchin embryos using different approaches, including morphologic/morphometric analysis, metal-related proteolytic activities, and metalloproteinases-like activities.

### 3.1. Morphological and Morphometric Variations Induced by V

In recent papers, we have described the morphological alterations caused by V during embryo development [3,22,23]. Here, for the first time, we have associated morphological and morphometric parameters. Several studies have shown that the volume of embryos represents a relevant measure to study their response, as it reflects further aspects related to embryos’ health state [55,56,57,58]. Morphology allowed us to identify, for each V concentration, at which time of treatment either a developmental delay or morphological anomalies appeared. In addition, the morphometric parameter provided us important information about the nutritional status of the embryos, the efficiency of biomineralization, and the respect of embryo architecture. Using an integrated analysis of all of these parameters, we can assert that the V-exposed embryos, in addition to delays and anomalies, are affected by a lack of volume increase, indicative of poor growth and the absence of anatomical structures, mainly the skeleton (Figure 1).

In embryo development, metal-related proteolytic activities are massively involved in the remodeling of the extracellular matrix. For this reason, the study of the anatomical parameters was of fundamental importance to provide an explanation for the enzymatic activities differently expressed during the physiological or V-induced development and their localization in specific embryonic districts.

Our data demonstrated that V-effects on morphological and morphometric parameters were dose-/time-dependent, and the embryos in the earliest stages of development (from fertilization to intermediate gastrula) resisted metal toxic insult.

The presence of this threshold time (21 h) value seems to be in agreement with what has been reported in the literature on the V-accumulation kinetics and on the intracellular Ca measurements. In these stages of embryonic development, the amount of accumulated V did not threaten the correct growth. The intracellular Ca amount remained comparable to control embryos (up to the stage of intermediate/advanced gastrula) [23].

### 3.2. V-Accumulation Is Related to Treatment Doses and Competes with Ca Uptake

Previous data about V-effects on skeleton (i.e., length and weight of spicules) suggested a competition between V and Ca [3] and confirmed that V accumulated continuously as development proceeded [23].

Here, we measured the total V and Ca amount incorporated by the embryos at the end of the entire treatment period (48 h). We noticed that, with the two V doses, the accumulated quantity was not exactly dependent on its concentration. Indeed, the amount of V accumulated in embryos treated with V 1 mM was not double that found in embryos treated with half a dose (V 500 µM).

One possible explanation is that embryos exposed to both V concentrations triggered a cell-selective apoptosis, sacrificing the excessively damaged cells in order to defend the development program [3]. As the percentage of apoptotic cells in 1 mM V-treated embryos was 28% higher than that of 500 µM V-treated embryos [23], this could mean that these apoptotic cells did not contribute to global V-accumulation. Indeed, the V-amount detected by ICP-MS for these 1 mM V-treated embryos was reduced by about 21% compared with the expected V-quantity (Figure 2A).

In the same way, the Ca amount in both V-treated embryos was much lower than the concentration detected in controls, confirming a competition between the two metals during the exposure time (Figure 2B). These data correlated with the lack of skeletal formation, as observed by morphological analyses.

### 3.3. Proteolytic Activity Correlates with V-Exposure

Embryo development is a complex pathway in which the growing organism continuously and quickly changes its anatomical, morphological, tissue, cellular, and molecular characteristics, and extracellular matrix reorganization plays a fundamental role in all these processes. The presence of at least nine metal-related proteolytic activities and their V-induced modulation has been already reported for embryos at the pluteus stage (36 h of development/treatment) [22].

Here, we tried to elucidate their global contribution to development, both under physiological growth condition and after V-exposure.

Total proteolytic activity was reduced in V-exposed embryos and this effect was dose-related. However, our results showed that V-treated embryos exhibited a tolerance threshold. When treated with gradually reduced V-concentrations and allowed to develop until pluteus (36 h), the embryos showed a global proteolytic activity comparable to controls (Figure 3D,E). These data correlate with our previous results. Indeed, already at the lowest V-concentrations, including those found in polluted environments, embryos were able to activate stress response strategies through the action of HSPs, autophagic mechanisms, and a cell-selective apoptosis. In this way, they try to safeguard the development program by eliminating only the damaged cells [3].

During physiological development (i.e., HB, gastrula, and pluteus stages), we have detected three peaks in the total proteolytic activity that are downregulated by V (Figure 3B). At HB, the high levels of total proteolytic activity could be attributed to the action of the hatching enzyme (envelysin). Indeed, about 11 h after fertilization, sea urchin embryos hatch out from the protective fertilization envelope [59]. During this hatching process, the activation of this protease takes place, which, because of its biochemical characteristics, could be considered a member of the mammalian MMP family [60]. Going forward in development (gastrula and pluteus), the increased total proteolytic activity is associable with the intense cell migration and tissue movements, markedly active in these phases [22,30].

### 3.4. Single Proteolytic Activities Are Differently Modulated by V

The different proteolytic activities of each of the nine detected gelatinases could suggest different roles in physiological development and after V-treatment.

In the latter case, a reduction or an increase in the single proteolytic activity could indicate either a consequence of the damage induced by the metal or, alternatively, a defense strategy activated by the embryos. In our opinion, as the degradation activities of the extracellular matrix are at the basis of the complex dynamics that govern cell and tissue movements in embryos, their different expression probably correlates with a new adaptation phenotype. Therefore, in our analysis, an increase in proteolytic activity could represent a defense strategy aimed at establishing a new developmental phenotype, whereas a reduction could suggest an extremely stressful condition.

In control embryos, all nine gelatinases were present during embryonic development. In particular, in the early stages of embryogenesis (12–24 h), there was a high level of gelatinase activity mediated by the low molecular weight proteases (34, 30, 25, and 22 kDa). On the contrary, in the later developmental stages (30–48 h), there was a high level of gelatin activity mediated by high molecular weight proteolytic activities (309, 255, 177, 79, and 59 kDa) (Figure 4).

In V-exposed embryos, the 309 and 255 kDa gelatinases were likely indicative of a specific dose-induced response of V, as their levels were more elevated in embryos exposed to 1 mM than in 500 µM ones (Figure 4(A2,B2)). This dose dependence suggests that a higher V-concentration increased these proteases as a consequence of the stressful situation.

V-exposed embryos could prefer the action of these two enzymatic activities as a protective strategy, further activating the remodeling of the extracellular matrix, in an attempt to establish a new adaptation phenotype.

The 177, 79, and 59 kDa gelatinase showed a high level of activity in embryos exposed to the lowest dose of V (500 µM) compared with embryos exposed to the highest concentration (1 mM) (Figure 4(C2,D2,E2)). Although both embryo types carry out a cell-selective apoptosis to eliminate excessively damaged cells, there was a reduction in mortality of 28% in embryos exposed to 500 µM V compared with embryos exposed to V 1 mM, which appeared less viable [3,23]. This could suggest that the former dose could induce greater enzymatic activity as a defense strategy triggered by embryos, justifying the peaks observed for these proteases. In the case of the 79 kDa gelatinase, 500 µM V-treated embryos showed two peaks in two different developmental stages (30 h and 42 h). In the same way, the 59 kDa protease also showed two peaks, but in two different developmental stages (30 h and 48 h) (Figure 4(D1,E1)). In both cases, these peaks could represent the stages of major adaptation to the stressful situation. Finally, 177 kDa showed a constant increase in its levels during development, reaching the maximum at 48 h (Figure 4(C2)).

The presence of these peaks in those developmental stages in which skeletogenesis should occur could explain the reasons that skeletogenesis was absent in embryos exposed to 1 mM and inhibited in embryos exposed to 500 µM. It is possible to hypothesize that the processes underlying the biomineralization (remodeling of the matrix) remain active over time, even if the formation of calcareous spicules was not observed [3].

On the other hand, the group of low molecular weight gelatinases (34, 30, 25, and 22 kDa) has a more homogeneous behavior. In all cases, the embryos exposed to both V-concentrations tested showed a reduction in the levels of these enzymatic activities compared with the control (Figure 4(F2,G2,H2,I2)).

### 3.5. Metalloproteinases-2-, -9-, and -14-Like Identification and V-Induced Specific Modulation

In our previous paper, we demonstrated that proteases were important in *P. lividus* embryos both in physiological development and after V-treatment. Biochemical characterization suggested that some of them could be MMPs [22]. These enzymes are required for several physiological processes, such as reproduction, morphogenesis, embryo development, bone remodeling, angiogenesis, and tissue repair, but they can also contribute to tissue destruction during cancer development and spreading, to arthritis/osteoarthritis, and to fibrotic diseases [35].

Genome analysis of the sea urchin *Strongylocentrotus purpuratus* revealed approximately 240 metalloprotease genes, representing all 23 families expressed in vertebrates. In addition, the size of the sea urchin MMP family and the clustered arrangement of many of its members are similar to those of vertebrates, but phylogenetic analyses suggest that different ancestral genes were independently amplified in sea urchins and vertebrates [30].

In this paper, we have identified MMP-2-, -9- and 14-like proteins, and we have studied their involvement in both physiological sea urchin development and following V-treatment.

In particular, although multiple MMP-like isoforms could be involved, we focused our attention on the MMP-14-like protein, due to its key role during embryonic development. Previous studies have highlighted its pleiotropic functions, especially in the regulation of developmental events requiring extracellular matrix remodeling, and only a few studies suggested its role in cell polarity determination and cell migration in embryo development [36,41]. Inhibition of MMP-14 metalloproteinase activity is known to block neural crest cells migration, while contributing to the formation of the derivative structures of these cells during embryogenesis [52]. Moreover, loss of MMP-14 function leads to severe skeletal abnormalities in mouse [37,49,50,51].

Our results on the MMP-14-like protease suggest that it could be involved in the modulation of V response. During physiological development, MMP-14 was detected at high levels from HB to AG (Figure 5D). In this phase, there is no intense skeletogenic activity, so its high levels could be necessary for the proteolytic action on the organic extracellular matrix, before the inorganic (calcareous) one’s deposition. The AG stage represents the switch in which the amount of intracellular Ca begins to increase significantly compared with the previous embryonic stages, where intracellular Ca concentration remains almost constant [23]. In the subsequent development stages, a parallel increment in intracellular Ca and deposition of inorganic matrix (especially CaCO_3_) was reported [3,23]. These phases were mainly characterized by the synthesis of the calcareous skeleton (inorganic extracellular matrix) and were characterized by a decrease in MMP-14-like protein levels (marker of degradation of organic extracellular matrix). In fact, the physiological levels of the MMP-14-like protein were drastically reduced at the Pr stage and almost no signal was detected at the two APl stages (42 h and 48 h) when the skeleton was definitively complete (Figure 5D). Therefore, the MMP-14-like protein would seem to act before the deposition of the calcareous extracellular matrix.

Consequently, in embryos exposed to both V-doses (1 mM or 500 μM), the MMP-14-like levels were always high compared with control embryos, especially from 30 h to 48 h of exposure. As there was no calcareous matrix deposition, the embryos continued to express the MMP-14-like protein in a dose-dependent manner (Figure 5D).

Moreover, a continuous expression of the MMP-14-like enzyme could be one of the factors responsible for the attempt of the embryos to counteract environmental stress, in accordance with what has been reported for different types of chemical and/or phisical environmental stresses [61].

### 3.6. MMP-14-Like Protease Localizes in Specific Tissue Districts

In situ immunofluorescence assays were carried out to better characterize the role of the MMP-14-like protease, probably involved in the degradation of the organic extracellular matrix before the deposition of the calcareous one. In particular, we focused our attention on a development stage with an active skeletogenesis (i.e., Epl 36 h of development).

In control embryos, where only an increase in the calcareous mass is expected and an excessive remodeling of the organic matrix is not necessary, a weak fluorescence signal was detected (Figure 6(A1–A4)).

On the contrary, V-treated embryos showed high levels of the MMP-14-like protein (Figure 6(B1–C4)). This result was in accordance with the morphological data (Figure 1(G1–G3)) and with our previous quantitative data about Ca accumulation [23] and the deposition of calcareous material [3]. All these processes require a continuous remodeling of the extracellular matrix. In treated embryos, in addition to the high levels of expression of the MMP-14-like protease, its localization is also peculiar. Indeed, its signal localized on the cytoplasmic expansions (filopodia) produced by the skeletogenic cells (PMCs) (Figure 6). Similarly, this enzyme has already been observed in filopodia, lamellipodia, and invadopodia of migrant cells [33,44].

## 4. Materials and Methods

### 4.1. Embryos Cultures, V-Treatments, and Incorpoted Metals’ Determination

Adult specimens of *Paracentrotus lividus* sea urchins were harvested from cost of the Favignana Island MPA (Marine Protected Area), Sicily.

After fertilization, embryos were maintained in glass containers with gentle-mixing in natural Millipore filtered sea water (MFSW). Aliquots of embryo cultures were maintained as control, while other aliquots were cultured for different times and treated with several concentrations of sodium orthovanadate (Na_3_VO_4_, hereafter V). The different V concentrations were obtained from a stock solution (0.1 M) prepared to avoid the presence of decavanadate and to ensure the presence of vanadate monomers. In particular, salt was dissolved in distilled water adjusted to pH 10. This solution appeared yellow/orange as a result of decavanadate presence. To ensure the presence of vanadate monomers, the solution was boiled until translucent and the pH was readjusted to 10. The absence of decavanadate in the solution was confirmed by reading the stock solution (1:100 diluted) from 220 to 700 nm, by a Cary 100 UV/Visible Spectrophotometer (Repligen, Waltham, MA, USA).

The correspondence of nominal versus analytical V-concentrations used in this study and V and Ca quantitative analysis in embryos were determined by a set of ICP-MS analyses using an Aurora M90 Brucker apparatus.

### 4.2. Morphological and Morphometric Analysis of Sea Urchin Embryos

Morphological analyses of control and V-exposed embryos were obtained using a 10× objective of a BX50 Olympus microscope (Tokyo, Japan). Several images were captured through a Nikon Sight DS-U1 digital camera (Tokyo, Japan). About 100 embryos for each control and V-treated samples were analysed according to criteria defined elsewhere [62]. The results were expressed as a percentage of scored embryos (100 embryos for each sample).

Morphometric analyses were conducted in order to estimate the embryonic volume. For this purpose, control and V-treated embryos at the blastula, gastrula, and prism stage were approximated to a sphere, and the volume was calculated according to the following formula: V = 4/3 πr^3^, after measuring the mean radius (r). Control embryos at the pluteus stage were approximated to a square-based pyramid and the volume was calculated according to the formula V = (Ab × h)/3 (A = base area; h = pyramid height). Therefore, for these embryos, the side of the base (average distance between the apex of the pre-oral arm and apex of the post-oral arm) and the height (distance between the center of the base and the apical region) were measured.

### 4.3. Polyacrylamide Gel-Based Gelatin Zymography

Pellets from control and V-treated embryos at different times were obtained from cultures by centrifugation at 1000× *g*. Lysis was carried out in a buffer (20 mM Tris, pH 7.4; 150 mM NaCl; 0.5% Triton X-100) without any type of protease inhibitors. Samples were lysed by three cycles of freezing (liquid nitrogen) and thawing (37 °C water bath).

Cell debris were removed by centrifugation at 12,000× *g* for 30 min at 4 °C and supernatants were stored at −80 °C until used. Then, 15 µg of proteins for each sample, determined by Bradford method with BSA as a standard, was separated by 10% SDS-PAGE gel zymography and treated, as described by Chiarelli et al. [22].

Gelatinase bands were quantified using the Quantity One software (Bio-Rad, Hercules, CA, USA).

### 4.4. Electrophoretic Analysis and Immunoblotting

Pellets were recovered from 5 mL of embryo cultures at 12, 15, 18, 21, 24, 30, 36, 42, and 48 h of development and were homogenized using a lysis buffer (7 M CH_4_N_2_O, 2% CHAPS, 10 mM C_4_H_10_O_2_S_2_), as described by Chiarelli et al. [23].

The Bradford method with BSA as a standard was used to determinate the protein concentration and 30 μg of extracted proteins was separated by 10% SDS-PAGE.

After electrophoresis, proteins were transferred to 0.45 μm nitrocellulose blotting membranes and reacted with the following primary antibodies: MMP-2 (Genetex, cod. GTx30147, Irvine, CA, USA); MMP-9 (Biorbit, cod. 11064, Cambridge, UK); MMP-14 (Biorbit, cod. ORB 11058); and actin (Sigma-Aldrich, cod. A5060, Waltham, MA, USA), according to the following dilution: 1:1000; 1:250; 1:250; and 1:500, respectively, in 5% Blotto non-fat milk/TBS-T (Tris-Buffered Saline, 0.1% Tween^®^ 20). The used secondary antibodies were anti-rabbit IgG HRP conjugate (Promega, cod. W401B, Madison, WI, USA) or anti-mouse IgG HRP conjugate (Promega, cod. W402B), dilution: 1:2500.

Protein immunoreactive bands were identified using the Immun-Star™ Western C™ chemiluminescent kit (Bio-Rad) according to the manufacturer instructions through the Molecular imager VersaDoc MP Systems (Bio-Rad).

Quantity One software (v.4.6.6, Bio-Rad) was used to quantify band intensities, referring to the actin bands as a loading control.

### 4.5. Anti-MMP-14 and Confocal Laser Scanning Microscope Analysis

Immunofluorescence was performed on whole-mount control and V-treated embryos fixed, as previously described by Chiarelli et al. [11]. Subsequently, each sample was incubated for 1 h at room temperature in blocking solution: 0.5% albumin from bovine serum (Sigma-Aldrich, A7906) and 5% heat inactivated goat serum (Sigma-Aldrich, G9023) in PBS-T (phosphate-buffered saline, 0.1% Tween^®^ 20) and overnight at 4 °C with anti-MMP-14 antibody, 1:100 diluted in blocking solution. In the negative control, the primary antibody was omitted. After rinsing with PBS-T, embryos were incubated with an Alexa Fluor^®^ 488 goat anti-rabbit IgG (H+L, Bristol, UK) secondary antibody (Molecular Probes, cod. A11008, Eugene, OR, USA), 1:250 diluted in blocking solution. The nuclei were stained with propidium iodide (0.18 µg/mL) for 5 min. Samples were mounted on glass slides in 80% glycerol/PBS-T, covered with a coverslip, and observed by confocal laser scanning microscope (CLSM) (Olympus FV10i). Several optical sections of embryos (10 µm thick) were captured. The intensity of autofluorescence emitted by the negative control was used as a threshold for the other samples.

### 4.6. Statistical Analysis

Percentage data for each of the morphological categories were analysed by two-way analysis of variance (ANOVA) with time and V concentrations as fixed factors. The percentage data were arc sin square root transformed prior to analysis. Tukey’s HSD test was used as a post-hoc test for mean comparison. Homogeneity of variance was checked and confirmed using Levene’s test. The analyses were performed using the Statistica 13.2 software (StatSoft, Tulsa, OK, USA) with *p* < 0.05 as the level of significance.

Values of morphological and morphometric analysis, gelatinase activities, and immunoblot assay were carried out on embryos obtained from three independent fertilizations and were analysed by unpaired two-tailed Student’s *t*-test. The analyses were performed with GraphPad Prism 9 software (GraphPad). The statistical significances were set to *p* ≤ 0.05 (*), *p* ≤ 0.01 (**), and *p* ≤ 0.0005 (***).

All data are represented as means of three independent experiments (*n* = 3) ± standard deviation (SD).

For morphological/morphometric analyses, 100 randomly collected embryos from each batch were sampled for microscopic examination. The datum reported and used for statistical analysis is the mean of 100 scored embryos per each independent experiment [63], thus *n* = 1 corresponds to 100 embryos for each experimental condition.

For ICP-MS, 250,000 embryos were used for each experimental condition.

## 5. Conclusions

In conclusion, we showed that V-exposure affects *Paracentrotus lividus* embryos at different levels: molecular, cellular, tissue, and anatomical. Morphological aberrations mainly concerned the correct distribution of embryonic cells and tissues and the development of the skeleton. These affect the size of embryos, which in turn provides important information on the embryos’ trophic state, strongly disturbed by V.

V, as already reported [22], induced an imbalance in the concentrations of other metal ions and this influences all the metal-dependent mechanisms present at the cellular level. In particular, many enzymes that use metals as a cofactor could be modified. Indeed, throughout the development/treatment, V promoted a modulation of the proteolytic activity of gelatinolytic enzymes. Among the various involved proteolytic enzymes in physiological and V-induced development, MMPs play a key role [22,35]. We have demonstrated the presence of three MMP-like gelatinases in the sea urchin (MMP-2, -9 and -14). In particular, we monitored the expression levels of the MMP-14-like protein during the entire development/treatment period. Moreover, the localization of this enzymatic activity, especially at the level of the filipodia, suggests that this protease could be actively synthesized by the PMCs.

Overall, these enzymes could be adequate markers in the study of the effects induced by V and, together with the study of the other gelatinases, could easily reflect the modulation induced by this metal.

Our data could also contribute to increasing the knowledge on the strategy activated by sea urchin embryos against chemical stress.

## Figures and Tables

**Table 1:** Frequency of embryonic stages detected at different V-concentrations and different developmental times. Developmental stages are indicated as HB (hatching blastula); MB (mesenchyme blastula); EG (early gastrula); IG (intermediate gastrula); AG (advanced gastrula); Pr (prism); EPl (early pluteus); and APl (advanced pluteus). * Altered phenotypes. Data are presented as the mean of triplicate experiments (*n* = 3).

Developmental Time	V (M)	HB (%)	MB (%)	EG (%)	IG (%)	AG (%)	Pr (%)	EPl (%)	APl (%)
12 h	-	100							
	1 mM	100							
	500 µM	100							
15 h	-		100						
	1 mM		100						
	500 µM		100						
18 h	-			100					
	1 mM		3	97					
	500 µM			100					
21 h	-				100				
	1 mM			98	2				
	500 µM			50	50				
24 h	-					100			
	1 mM			8	90	2			
	500 µM			50		50			
30 h	-						100		
	1 mM				98	2			
	500 µM				50	50			
36 h	-							100	
	1 mM					100 *			
	500 µM					98 *	2		
42 h	-								100
	1 mM					100 *			
	500 µM					98 *	2 *		
48 h	-								100
	1 mM					100 *			
	500 µM					98 *	2 *		

## Data Availability

Not applicable.
